# An effectiveness study of an integrated, community-based package for maternal, newborn, child and HIV care in South Africa: study protocol for a randomized controlled trial

**DOI:** 10.1186/1745-6215-12-236

**Published:** 2011-11-01

**Authors:** Mark Tomlinson, Tanya Doherty, Debra Jackson, Joy E Lawn, Petrida Ijumba, Mark Colvin, Lungiswa Nkonki, Emmanuelle Daviaud, Ameena Goga, David Sanders, Carl Lombard, Lars Åke Persson, Thoko Ndaba, Gail Snetro, Mickey Chopra

**Affiliations:** 1 Department of Psychology, Stellenbosch University, Stellenbosch, Private Bag X1, Matieland, 7602, South Africa; 2 Health Systems Research Unit, Medical Research Council, Francie van Zijl Drive, Parowvallei, PO Box 19070, 7505 Tygerberg, South Africa; 3 School of Public Health, University of the Western Cape, Private Bag X17, Bellville, South Africa; 4 Saving Newborn Lives, Save the Children, 11 South Way, Cape Town 7405, South Africa; 5 Maromi Health Research, 27 Cohen Avenue, Glenwood, 4083, South Africa; 6 Biostatistics, Medical Research Council, Francie van Zijl Drive, Parowvallei, Cape;PO Box 19070, 7505 Tygerberg, South Africa; 7 International Maternal and Child Health, Uppsala University, University Hospital, SE-751 85 Uppsala, Sweden; 8 Chief of Health, UNICEF, 3 UN Plaza, New York, NY 10017, USA

## Abstract

**Background:**

Progress towards MDG4 in South Africa will depend largely on scaling up effective prevention against mother to child transmission (PMTCT) of HIV and also addressing neonatal mortality. This imperative drives increasing focus on the neonatal period and particularly on the development and testing of appropriate models of sustainable, community-based care in South Africa in order to reach the poor. A number of key implementation gaps affecting progress have been identified. Implementation gaps for HIV prevention in neonates; implementation gaps for neonatal care especially home postnatal care; and implementation gaps for maternal mental health support. We have developed and are evaluating and costing an integrated and scaleable home visit package delivered by community health workers targeting pregnant and postnatal women and their newborns to provide essential maternal/newborn care as well as interventions for Prevention of Mother to Child Transmission (PMTCT) of HIV.

**Methods:**

The trial is a cluster randomized controlled trial that is being implemented in Umlazi which is a peri-urban settlement with a total population of 1 million close to Durban in KwaZulu Natal, South Africa. The trial consists of 30 randomized clusters (15 in each arm). A baseline survey established the homogeneity of clusters and neither stratification nor matching was performed. Sample size was based on increasing HIV-free survival from 74% to 84%, and calculated to be 120 pregnant women per cluster. Primary outcomes are higher levels of HIV free survival and levels of exclusive and appropriate infant feeding at 12 weeks postnatally. The intervention is home based with community health workers delivering two antenatal visits, a postnatal visit within 48 hours of birth, and a further four visits during the first two months of the infants life. We are undertaking programmatic and cost effectiveness analysis to cost the intervention.

**Discussion:**

The question is not merely to develop an efficacious package but also to identify and test delivery strategies that enable scaling up, which requires effectiveness studies in a health systems context, adapting and testing Asian community-based studies in various African contexts.

**Trial registration:**

ISRCTN: ISRCTN41046462

## Background

South Africa has been spotlighted as one of only a dozen countries worldwide where child mortality has increased since the baseline for the Millennium Development Goal 4 (MDG4) for child survival was set in 1990 [1]. This is primarily related to the HIV epidemic and more than half of the child deaths are attributed to HIV/AIDS, but much less attention is given to the fact that 30% of under five deaths in South Africa are neonatal - deaths in the first 28 days of life [2]. Progress towards MDG4 in South Africa will depend largely on scaling up effective prevention against mother to child transmission (PMTCT) of HIV and also addressing neonatal mortality. This same time period is critical for maternal survival and health (MDG5). This imperative drives increasing focus on the neonatal period and particularly on the development and testing of appropriate models of sustainable, community-based care in South Africa in order to reach the poor, especially the peri-urban poor. A number of key implementation gaps affecting progress have been identified [2-4].

### 1. Implementation gaps for HIV prevention in neonates

South Africa is one of the highest HIV prevalence countries in the world and has the single largest number of people living with AIDS. Although the HIV prevalence appears to be levelling off at around 30% of pregnant women, this means that around 300, 000 women and their babies require identification, support and appropriate care [2]. Reducing MTCT of HIV is a major policy and programmatic priority and with increasing political support, more investment has focused on implementing effective ARV prophylaxis regimes. As is the case in much of Africa, coverage of PMTCT services in South Africa is sub optimal. Although HIV testing of pregnant women has increased steadily from 49% in 2005 to 86% in 2008, less than half of HIV positive pregnant women receive CD4 testing and coverage of infant six week PCR testing is even lower [5]. However appropriate infant feeding practices around birth and the first few weeks of life are also critical and as yet success is elusive. MTCT may occur during breastfeeding but the greatest risk is with mixed feeding. In South Africa exclusive breastfeeding rates are 7% [6]. In Umlazi data from the Good Start study found that less than half (46%) of HIV-positive women were able to practice either exclusive formula feeding or exclusive breastfeeding. Of the HIV-positive women who intended to EBF, less than 2% practiced EBF. These results are similar to those in rural South Africa [7]. The commonest reason given for introducing additional fluids during the first 48 hours of life was "insufficient breastmilk". From week one to week 16, additional fluids and semi-solids were given for an "unsatisfied baby" or "perceived milk insufficiency". It has been shown that intensive counselling and support can increase the proportion of women who breastfeed exclusively [8-10]. However there are considerable variations in the type of counselling support provided and many of these interventions were very intensive and unlikely to be scaleable within an overstretched health system, particularly if the peer counsellor's sole responsibility is feeding support and not additional tasks. Disclosure of HIV status remains a major constraint in effective counselling and behaviour change.

### 2. Implementation gaps for neonatal care especially home postnatal care

Globally neonatal deaths are an increasing proportion of under-five deaths [3]. Despite considerable gains in improving global child health, and in reducing child mortality after the first month of life (month 2 to age 5 years), the same gains have not been made in reducing neonatal mortality. Now 41% of all deaths in children under five occur in the neonatal period [11]. The neonatal mortality rate in South Africa is 21 per 1000 live births [2], although this differs markedly across regions in South Africa, and for the poorest. A baby in Western Cape may have access to full intensive care, yet a baby in rural Eastern Cape may not receive basic care. The newborn health component of maternal and child health programmes has been a missing link, and a continuum of care between homes and the health care system is key for newborn care practices and survival [12]. An increasing number of studies in South Asia have examined community based packages for newborn care, reporting reductions in neonatal mortality of up to 70% [13-15], but as yet no such studies have been published from Africa, and none that also included PMTCT. The United Nations recently released a joint statement on the importance of home postnatal care for mothers and newborns [16], yet South Africa does not have such a package, or as yet a worker to provide such care.

### 3. Implementation gap for maternal mental health support

A third implementation gap at the time of birth in low and middle income countries is the dearth of interventions aimed at improving maternal mental state and addressing the effects that this has on child development [4]. Several recent studies have provided data on how maternal depression impacts infant physical growth, how improved mother-infant interaction improves breastfeeding and infant and child development, and finally, a recent randomized controlled trial has provided evidence of the benefits of an early community based intervention with mothers and their infants [17]. An intervention aimed specifically at preventing or treating maternal depression may not be cost effective in a developing world context, although Rahman and colleagues have shown the benefits of a community based intervention targeting depressed women [18]. This intervention however is embedded within the tasks of an existing cadre of Lady Health Visitors who deliver a series of health interventions in Pakistan. There is a possibility however, that the delivery of messages about maternal mood within an integrated package of home visits, together with the supportive counselling aspect provided by the CBHW's, may impact maternal mood.

#### Scientific rationale for integrated package to be tested

**Figure 1:**
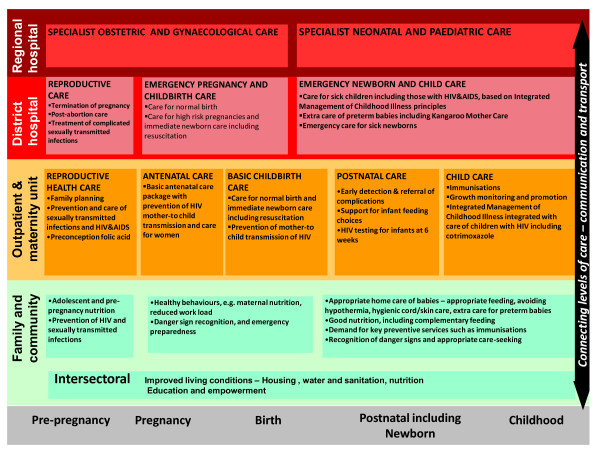
**Integrated healthcare packages for maternal, newborn and child health according to level of the health system in South Africa**. Source - Chopra et al [2] adapted from Kerber et al [19].

There is an urgent need to develop and implement an evidence based, cost-effective package that can be delivered on a wide scale including reaching the poorest families, in order to achieve key linked outcomes around the time of birth - reducing neonatal mortality, preventing mother to child transmission of HIV, and impacting maternal mood. Integration of HIV/AIDS PMTCT programmes with neonatal and childcare packages would contribute to improved HIV free survival, maternal health and also promote more efficient and effective use of resources in this key time period. A review of a variety of integrated service delivery packages has previously suggested 8 integrated MNCH packages which also include PMTCT of HIV [19], and this global set has been adapted for national application in South Africa to also include the referral level (Figure 1) [2].

**Figure 2:**
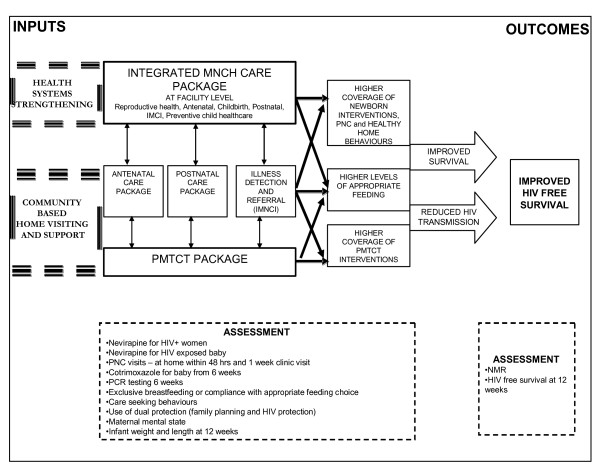
**An integrated community based MNCH and PMTCT package to improve HIV free survival**.

Our focus is on the community and primary care level, and on the time period of pregnancy, birth, the postnatal period and early weeks of life. Figure 2 illustrates our conceptualisation of this integrated maternal, newborn and child health care package and the relevant outcomes. It is envisaged that the MNCH package (antenatal, postnatal and illness detection and referral elements) will improve access to and uptake of PMTCT which in turn, either directly or indirectly, will lead to higher levels of appropriate feeding. The outcome of this will be improved HIV free survival.

Our study seeks to address gaps in previous research undertaken in South Africa namely a focus on the neonatal period and linking community based care with primary health care services. The first week after birth is the period during which most neonatal deaths (as well as most maternal deaths) occur and which is also most crucial in preventing MTCT. Interventions that better link the household and community level, and which bring skilled community health workers closer to communities have the potential to not only make a significant contribution to reducing mortality, but also to empowering communities. In many countries there is renewed interest in strengthening community based care and in evaluating what can be achieved for maternal, newborn and child survival at community level [20]. Effective counselling at home has been found to be more powerful than "health education" in a clinical setting [21]. The question is not merely to develop an efficacious package but also to identify and test delivery strategies that enable scaling up, which requires effectiveness studies in a health systems context, adapting and testing existing models in the African context. Intervention strategies need to be based in evidence as well as being culturally appropriate [22].

#### Policy context

The South African government is committed to meeting the MDG 4 target and were PMTCT and newborn care to be scaled up at full coverage this would put South Africa on target [2]. The South African National Department of Health (SANDOH) has also acknowledged that in order to increase the likelihood of meeting the health MDGs, the health system needs to be strengthened through re-engineering of the current PHC approach. Lessons learnt from the success of Brazil's Unified Health Programme/Family Health Programme, has led to the revision of the SANDOH PHC service package in 2010. The PHC approach will change from a passive, curative-oriented and based on individual health care to a proactive population-based approach utilising community based health workers (CBHWs) [23].

One major component of this new PHC plan is to employ CBHWs as generalist health workers. While community workers in this study are part of a research study rather than government hired and trained generalist community workers, every effort has been made to ensure that all aspects of the training, implementation and supervision of the community workers approximates interventions currently being planned by the South African government. In addition, the package promotes linkages between community level and existing health systems.

## Methods

### Study aim and objectives

The goal of this study is to develop, evaluate and cost an integrated and scaleable home visit package delivered by community health workers targeting pregnant and postnatal women and their newborns to provide essential maternal/newborn care as well as interventions for Prevention of Mother to Child Transmission (PMTCT) of HIV using a cluster randomized controlled trial design. We hypothesise that mothers and infants in intervention clusters will have

○ Higher levels of HIV free survival and levels of exclusive and appropriate infant feeding at 12 weeks postnatally

○ Better uptake of a postnatal clinic visit within 7 days of life

○ Better coverage of care and behavioural indicators (antenatal HIV testing, uptake of cotrimoxazole amongst HIV exposed infants at 6 weeks, family planning uptake at 6 weeks)

Secondary objective:

○ To assess post intervention levels of maternal depression at 12 weeks postpartum

### Study context

The site for the intervention is Umlazi which is a peri-urban settlement with a total population of 1 million close to Durban in KwaZulu Natal that has a mixture of formal and informal housing. The infant mortality rate is around 60/1000 live births, while there is no reliable figure of the neonatal mortality rate, most estimates place the NMR at about 25/1000 live births. The HIV prevalence amongst antenatal clients in this district was 41% in 2009 [24] Over 98% of all births in Umlazi occur in one major hospital.

### Study Design

#### Cluster selection and cluster size

The trial consists of 30 randomized clusters (15 in each arm). Maps were obtained that divided the area into "sub-places". Following the drawing of clusters a baseline survey of all clusters was conducted in order to check the homogeneity of clusters. Results of this exercise established the homogeneity of clusters and neither stratification nor matching was performed.

### Sample and sample size

The study sample is all pregnant women and their newborns in the cluster during the recruitment period who give informed consent for study participation. Sample size was calculated based on increasing HIV-free survival from 74% to 84%, with 80% power in an individually randomised trial (this would require 279 live births per arm). Assuming an ICC of 0.04 for a cluster randomised trial calculated that we would need 750 HIV exposed children per arm (50 per cluster) with a design effect of 3. Loss to follow up of approximately 20% was added to this sample size. Based on an HIV prevalence rate amongst pregnant women of 40% our sample size was calculated to be 120 pregnant women per cluster.

### Study procedures

#### Recruitment

In intervention clusters, CBHW's identify pregnant women and encourage them to seek antenatal care. CBHW's also inform pregnant women about the study that is being conducted on infant feeding and child health. In control clusters a CBHW recruits all pregnant women. CBHW's in control clusters provide key information and support to the mother on how to obtain social welfare grants, particularly the Child Support Grant. The grant package in the control arm comprises one visit during the antenatal period to provide the necessary information regarding requirements for a grant application. As these grants normally take a few months to be processed, there is a visit at 4-6 weeks postnatally to follow up the grant application and to assist in resolving any difficulties. There is also a visit by CBHW's in the control arm at 10-12 weeks with the main aim of reminding women to go to the assessment site when their infant is 12 weeks old for data collection.

The size of intervention and control clusters are the same. All efforts have been made to avoid contamination between control and intervention arms. The focussed, one on one, and individualised nature of the intervention in itself, significantly limits the possibility of contamination. On a weekly basis the data collectors are given the cards of women identified by the CBHW's. They go to the mother's homes to inform them about the study in detail and obtain written, informed consent from mothers who wish to participate in data collection. They ask mothers if they underwent counselling and HIV testing at the antenatal clinic and ask if the mother is willing to disclose her test result. The activities of the CBHW's and data collectors are kept completely separate.

In order to ensure that CBHW's are able to visit women in the first 24 hours following birth we have employed a part-time person in the Prince Mshiyeni hospital where over 98% of all births in Umlazi take place. The person obtains daily records of all births in the Prince Mshiyeni hospital, and contacts (via the mobile phone system and web interface) CBHW's when an intervention group participant gives birth.

#### Intervention

CBHW's were trained in the intervention over the course of two weeks. The training included the extensive use of role plays that were video recorded and then used for teaching and supervision purposes. The intervention is a structured home visiting programme with antenatal and postnatal visits with specific content covered at each visit. These community level interventions along the continuum of care link to the existing health system packages delivered through outreach (antenatal, some postnatal visits and preventive child health care and clinical care at facilities including obstetric and emergency newborn and child care). In each intervention cluster there is one CBHW who covers all households in the cluster. The content and conduct of the intervention is specified in a manual which is used to train community workers, linking to existing PMTCT, Integrated Management of Childhood Illness (IMCI) and newborn care guidelines. There are various linkages between all the programmes mentioned above that aid the process of programme integration.

The visits in the intervention arm are as follows (see Table 1 for visit content):

**Table 1:** Visit schedule and content

**Antenatal visit 1 at HOME**	Antenatal care action - immunisations/micronutrient supplementation Focus on the importance of VCT (linking this with the PMTCT programme and the benefits of testing to the mother) Emphasise the importance of antenatal care Key messages on appropriate infant feeding Encourage exclusive breastfeeding in HIV negative women or women of unknown HIV status. For HIV positive women, assist with thinking about infant feeding options Input regarding infant communication and the mother-infant relationship
**Antenatal visit 2 at home**	Birth plans - place of birth, support during labour, care plans if returning to work Danger signs and emergency plans - this will be done, if possible together with other family members in order to elicit their input regarding possible plans in the event of an emergency, including recognition of danger signs, emergency transport plan and emergency funds if needed Homecoming arrangements Follow up and re-emphasis on VCT, PMTCT, the key messages on appropriate infant feeding that were provided in antenatal visit 1; further discussion in terms of assisting with the implementation of chosen feeding option Additional input on infant communication and the warning signs of postnatal depression
**Postnatal visit 1 at home (24-48 hrs)**	Assessment of newborn-breathing, thermal care, colour, bleeding, neonatal eye care, checklist of danger signs Assessment of mother - bleeding, signs for infection, mastitis Early recognition of illness (superficial or systemic) and help seeking Exclusive breastfeeding or appropriate infant feeding support Hygienic cord care and what to expect regarding when the cord will drop off Thermal care, skin to skin care and Kangaroo care if needed for preterm babies Ensure that babies of HIV positive women have received Nevirapine Information about warning signs for mother or baby and what to do Support for women who have 'the blues'
**Postnatal visit 2 at home (3-4 days)**	Assessment of the mother and the newborn, Further input on the early recognition of illness (superficial or systemic) and help seeking Monitoring and follow up of breastfeeding or appropriate feeding and possible feeding problems Further support for hygiene, thermal care and cord care, with Kangaroo care input if needed for preterm babies 1^st ^week clinic visit reminder Information about warning signs for mother or baby and what to do Newborn Interactive Assessment - demonstration of the abilities of infants and infant communication and social responsiveness. Sleeping, crying, consolability and wider concerns
**Postnatal visit 3 at home (10-14 days)**	Early recognition of illness (superficial or systemic) and help seeking Ongoing monitoring of breastfeeding or other appropriate feeding Information about warning signs for mother or baby and what to do Promote attendance at clinic for 6 week visit for mother to have access to family planning and baby to receive immunisations and the babies of HIV+ women been given bactrim and HIV testing Mother infant interaction modelling and communication input Assess for signs of postnatal depression
**Postnatal visit 4 at home (3-4 weeks)**	Early recognition of illness (superficial or systemic) and help seeking Ongoing monitoring of breastfeeding or other appropriate feeding Information about warning signs for mother or baby and what to do Promote attendance at clinic for 6 week visit for mother to have access to family planning and baby to receive immunisations and the babies of HIV+ women been given bactrim and HIV testing Mother infant interaction modelling and communication input Assess for signs of postnatal depression
**Postnatal visit 5 at home (7-8 weeks)**	Further input on feeding including advice regarding weaning Infant weight from clinic card (6 week visit) Mother infant attachment Checklist of signs of postnatal depression Has the child been tested for HIV at six weeks and receiving cotrimoxazole Formula sustainability for HIV positive women using formula milk Family planning and counselling Input on milestones and information and specific skills about the stimulation of infants

○ Two antenatal visits

*○ Visit in the first 24-48 hours*.

○ Visit at day 3 or 4

○ Visit at 10-14 days

○ Visit at 3-4 weeks

○ There will then be a routine clinic visit at 6-7 weeks

○ Final visit at 7-8 weeks

All neonates that weigh under 2500 g that are released from hospital within 24 hours receive two extra visits. Neonates that weigh under 1500 g and when there are concerns about birth weight may be kept under observation at Prince Mshiyeni hospital. The discharge date of these neonates is considered as the date of birth for the purposes of the timing of visits. Extra time in hospital (more than 3 days) will be used as a marker for illness or low birth weight, and these mother infant dyads will receive an extra two visits during the first week at home.

#### Content of the visits

We have developed a package based on a review of existing evidence and taking the local context into account structured around a series of homes visits with specific tasks for each visit. These visits will also link to and promote visits in clinics and be timed to maximise uptake of the facility visits. CBHW's are provided with a book/flip chart book that contains details of material to be covered during visits. This is not designed as education material to be presented to participants but rather as a reminder to ensure that core messages are communicated during visits.

#### Health facility strengthening

A central component of the package is to promote linkages between community level and existing health systems. The Partnership Defined Quality (PDQ) approach works to reach marginalized populations and address underlying causes of health problems such as discrimination, social, economic, cultural, political and organizational conditions through addressing the quality of health services [25]. An integral part of this process involves providing community members and health facility providers with the skills and systemic support they need to improve health service quality and access to these services. The PDQ process is a collaborative approach to exploring health quality, identifying common issues and creating a common platform for communities and health service providers to work towards improving health quality service delivery. The PDQ process encourages and support communities and health services to actively participate together towards improving health quality through on ongoing dialogue, planning, collective action and monitoring of outcomes leading towards increased demand for and access to health services. We are implementing a PDQ process in two local clinics in Umlazi.

#### Data collection

Two types of data are consistently monitored:

1) *Medical record reviews *are conducted on the Road to Health card for each participant, extracting information on routine health data including HIV test results, CD4 count, tuberculosis, and PMTCT medications used, such as AZT and NVP. Delivery data, such as the date of delivery, baby weight and height, birth complications, and data from postnatal screening (vitamins dispensed, mother's TB and HIV test results, and the baby's weight, height, PCR test results, and immunisations) are also collected from the hospital delivery records.

2) *In-person interview assessments*. Structured interview guides have been developed for interviews with participants at 12 weeks postpartum. Interviews are administered by trained interviewers at the study office in Prince Mshiyeni hospital in Umlazi (see Table 2 for a list of process and outcome measures).

**Table 2:** Outcome measures

**Mortality, growth and HIV status**	Neonatal mortality rate (deaths in the first 28 days of life, per 1000 live births) Identification of HIV status in infants at 12 weeks using PCR testing Infant weight and length at 12 weeks
**Coverage of care**	Co-trimoxazole initiation amongst HIV exposed infants at 6 weeks Clinic visit within the 1^st^week Having received a postnatal care visit by a CBHW within 48 hours Assessment of biological markers of illness such as diarrhoea Assessment of uptake of HIV specific care and treatment for HIV positive families
**Key behaviours**	Infant feeding patterns will be recorded through 24 hour and previous three-day recall Participation in ANC, VCT, Well-child, Post-partum and PMTCT services Satisfaction with ANC, Delivery, Newborn, Post-partum and PMTCT services HIV status disclosure Maternal care behaviours of infant cleanliness, warmth Positive behaviours such as the occurrence of immediate (first hour of life) and exclusive breastfeeding at six weeks. Infant feeding patterns will be recorded through 24 hour and previous seven-day recall at 12 weeks. Maternal care-seeking behaviour for infant illness, detection of illness - recognizing signs and severity will be recorded at 12 weeks Use of dual protection methods for family planning and HIV/STD prevention recorded at 12 weeks Maternal mental state assessed at 12 weeks

In addition key process documentation will include an assessment of the process involved in training and supervising community workers, and the retention of CBHW's and factors affecting motivation and intensity of the intervention (coverage of scheduled visits).

#### Economic evaluation

Costing is a critical component of an effectiveness trial to inform scale up, and we are undertaking programmatic and cost effectiveness analysis. The economic evaluation is divided into the costing and cost-effective analysis (CEA). Both analyses will be conducted from a provider's perspective. The costs will be measured prospectively using a tool developed by two of the authors (ED and LN). The tool built on the gaps of existing tools [26]. The costing methodology will be an activity based costing using an ingredients approach [27-29]. The outputs of the intervention will be calculated (i.e. cost per visit and cost per woman).

In order to inform scale up process the costs will be presented as set-up costs (one-off and repeatable) and implementation costs. The use of the term scale up in this context refers to two processes; increasing coverage within the same district or geographical area and expanding coverage to other districts or geographical area. One-off and repeatable set up costs will be presented to highlight cost consequences for these different aspects of scale-up mentioned above. For both aspects of scale up one off and set-up costs will not be incurred again, examples of these costs are development of the content of the intervention and manual development. On the other hand, if the intervention is expanded to other districts, repeatable set-up costs will be incurred. These include recruitment and training of community health workers and their supervisors. If there is an existing cadre of community health workers and supervisors, and they have additional capacity (i.e. e time), recruitment costs (repeatable scale-up) and other implementation costs will not be applicable; this will translate to a reduction in unit costs (cost per visit). Another factor that affects scale-up is human resource requirements. In this trial the time use of community health workers has been captured in detail. This information will be useful establishing whether community health workers can take on additional activities or not. At facility level, recurrent cost-items consumed, and staff time will be measured. The purpose of this exercise will be to capture the consequences of the intervention at this level. CEA for the intervention will be undertaken based on findings from the costing studies and the trial described in this proposal. One of the trials primary outcome measures is HIV free survival at 12 weeks. Therefore, cost-effectiveness will be measured in terms of cost per Disability adjusted life year averted. The CEA will be stochastic to account for the level of uncertainty uncovered in the trial [30].

#### Data collection strategy

Mobile phones are used for collecting data at the 12 week endpoint as well as for monitoring the intervention delivery. Programming and bench testing of mobile phones for collection of assessment interviews was conducted for several months. Data is collected on mobile phones running a survey software package (Mobile Researcher; http://www.populi.net/mobileresearcher/). This platform allows the phone to be used to collect and upload numeric, voice and text data. The mobile phone models used in the study are the Nokia 5000 for data collection and 1680 for monitoring the intervention delivery. Initial training covers practical aspects of phone navigation, checking for software updates, use of the software, and uploading data to the central server. Staff then spent two weeks familiarising themselves with the baseline survey on the phone. During this time multiple tests were run to ensure that all data entered on the phone were uploaded and that the response entered on the mobile corresponded to the value stored in the database [31].

#### Determining HIV transmission rates

Dried blood spots (DBS) are collected from study infants of HIV infected mothers by means of a heel prick during the 12 weeks interview and tested using DNA PCR testing. The DBS samples are collected by Field Researchers who will have been trained in the procedure. Training included fully informing field researchers of the risks, the need to take universal precautions and on procedures related to accidental needle-prick injuries.

### Data analysis

Analyses will include descriptive and analytic statistics pertaining to research questions. Categorical data will be analysed using frequencies, cross-tabulations with chi-square and 95% confidence intervals for the risk ratio or risk difference. Continuous data will be analysed using means and variance, and comparison of mean differences and 95% confidence intervals around the mean difference. Kaplan-Meyer analysis will be used for estimating HIV transmission rates. Stratified analysis, logistic regression or Cox regression will be used to adjust for potential confounding in the data. Analysis will be on an intention to treat basis and we will ensure that the cluster randomized design will be accounted for in the analysis.

### Collaboration

The study is a collaborative effort between the Health Systems Research Unit, Medical Research Council (Tanya Doherty, Ameena Goga, Petrida Ijumba), Saving Newborn Lives/Save the Children (Joy Lawn), Stellenbosch University (Mark Tomlinson), University of Western Cape (Debra Jackson, David Sanders), Maromi Health Research (Mark Colvin), Uppsala University (Lars Ake Perrson) and UNICEF (Mickey Chopra).

### Research ethics and approval

The ethics review board of the Medical Research Council (EC08-002) has approved and monitors the study protocol over time. We have established a Community Advisory Board (CAB) that consists of a number of local stakeholders who act as a liaison between the community and research staff and management. The CAB also provides feedback to the community about study progress. Hospital and provincial authorities are also provided with regular feedback about the study.

## Discussion

The goal of the study is to develop an integrated and scaleable home visit package delivered by community health workers targeting pregnant and postnatal women and their newborns to provide essential maternal/newborn care as well as increasing uptake of interventions for Prevention of Mother to Child Transmission (PMTCT) of HIV. Integration of Prevention of Mother to Child Transmission of HIV/AIDS (PMTCT) programmes with maternal, neonatal and child health care packages to ensure reduction in mother to child transmission and promote more efficient and effective use of resources in this key time period is critical. A limitation of the study is the early outcome assessment at 12 weeks. Whilst this is a suitable time to collect outcomes related to newborn caring and early health care seeking, it will only allow for an assessment of early mother to child transmission and not later postnatal transmission which would require much longer and more costly follow up.

This study has a number of innovative aspects. Firstly this is an integrated package that is in line with a global focus on maternal and child health care but more importantly falls squarely within South African government policy of promoting integrated maternal, neonatal and child health. The mechanisms that have been put in place to ensure early (24-48 hours) home visits by CBHW's in the community is central to the reduction of neonatal mortality. Secondly, the use of mobile phones and a web-based interface aids in data collection but more importantly is proving to be an invaluable tool in the management and supervision of CBHW's.

## Abbreviations

AZT: Azidothymidine; CEA: Cost effective analysis; DBS: Dried blood spot; EBF: Exclusive breastfeeding; CBHW's: Community Based Health Workers; IMCI: Integrated Management of Childhood Illness; MDG: Millennium Development Goal; MTCT: Mother to child transmission; NVP: Nevirapine; PCR test: Polymerase Chain Reaction; PDQ: Partnership Defined Quality; PMTCT: Prevention of mother to child transmission; SANDOH: South African National Department of Health

## Competing interests

The authors declare that they have no competing interests.

## Authors' contributions

MT, TD, DJ, JL, MC, DS, LP, MC conceived of the study. MT, TD, DJ, JL, MC, DS, LP, MC, AG, ED, LN, CL, all contributed to writing the protocol for the study. All authors contributed to writing the paper. All authors have approved the final version of the paper.

## References

[B1] Bradshaw D Every death counts: use of mortality audit data for decision making to save the lives of mothers, babies, and children in South Africa Lancet 2008 371 9620 1294 304 18406864 10.1016/S0140-6736(08)60564-4

[B2] Chopra M Saving the lives of South Africa's mothers, babies, and children: can the health system deliver? Lancet 2009 374 9692 835 46 10.1016/S0140-6736(09)61123-5 19709729

[B3] Darmstadt GL Lawn JE Costello A Advancing the state of the world's newborns Bull World Health Organ 2003 81 3 224 5 12764520 PMC2572417

[B4] Rahman A Cognitive behaviour therapy-based intervention by community health workers for mothers with depression and their infants in rural Pakistan: a cluster-randomised controlled trial Lancet 2008 372 9642 902 9 10.1016/S0140-6736(08)61400-2 18790313 PMC2603063

[B5] Doherty T Day C, et al PMTCT Indicators The District Health Barometer 2008/09 2010 Health Systems Trust: Durban

[B6] Jackson DJ An update on HIV and infant feeding issues in developed and developing countries J Obstet Gynecol Neonatal Nurs 2009 38 2 219 29 10.1111/j.1552-6909.2009.01014.x 19323719

[B7] Bland RM Breastfeeding practices in an area of high HIV prevalence in rural South Africa Acta Paediatr 2002 91 6 704 11 10.1111/j.1651-2227.2002.tb03306.x 12162606

[B8] Morrow AL Efficacy of home-based peer counselling to promote exclusive breastfeeding: a randomised controlled trial Lancet 1999 353 9160 1226 31 10.1016/S0140-6736(98)08037-4 10217083

[B9] Barros FC The impact of lactation centres on breastfeeding patterns, morbidity and growth: a birth cohort study Acta Paediatr 1995 84 11 1221 6 10.1111/j.1651-2227.1995.tb13537.x 8580615

[B10] Kramer MS Promotion of Breastfeeding Intervention Trial (PROBIT): a randomized trial in the Republic of Belarus JAMA 2001 285 4 413 20 10.1001/jama.285.4.413 11242425

[B11] Lawn JE 3.6 million neonatal deaths--what is progressing and what is not? Semin Perinatol 2010 34 6 371 86 10.1053/j.semperi.2010.09.011 21094412

[B12] Martines J Neonatal survival: a call for action Lancet 2005 365 9465 1189 97 10.1016/S0140-6736(05)71882-1 15794974

[B13] Bang AT Effect of home-based neonatal care and management of sepsis on neonatal mortality: field trial in rural India Lancet 1999 354 9194 1955 61 10.1016/S0140-6736(99)03046-9 10622298

[B14] Kumar V Effect of community-based behaviour change management on neonatal mortality in Shivgarh Uttar Pradesh India: a cluster-randomised controlled trial Lancet 2008 372 9644 1151 62 10.1016/S0140-6736(08)61483-X 18926277

[B15] Baqui AH Effect of community-based newborn-care intervention package implemented through two service-delivery strategies in Sylhet district Bangladesh: a cluster-randomised controlled trial Lancet 2008 371 9628 1936 44 10.1016/S0140-6736(08)60835-1 18539225

[B16] WHO Home visits for the newborn child: a strategy to improve survival 2009 World Health Organization: Geneva 24809117

[B17] Cooper PJ Improving quality of mother-infant relationship and infant attachment in socioeconomically deprived community in South Africa: randomised controlled trial BMJ 2009 338 b974 10.1136/bmj.b974 19366752 PMC2669116

[B18] Rahman A Cluster randomized trial of a parent-based intervention to support early development of children in a low-income country Child Care Health Dev 2009 35 1 56 62 10.1111/j.1365-2214.2008.00897.x 18991970

[B19] Kerber KJ Continuum of care for maternal, newborn, and child health: from slogan to service delivery Lancet 2007 370 9595 1358 69 10.1016/S0140-6736(07)61578-5 17933651

[B20] Haines A Achieving child survival goals: potential contribution of community health workers Lancet 2007 369 9579 2121 31 10.1016/S0140-6736(07)60325-0 17586307

[B21] Chopra M Preventing HIV transmission to children: quality of counselling of mothers in South Africa Acta Paediatr 2005 94 3 357 63 16028656 10.1111/j.1651-2227.2005.tb03080.x

[B22] Hill Z Informed consent in Ghana: what do participants really understand? J Med Ethics 2008 34 1 48 53 10.1136/jme.2006.019059 18156522

[B23] SANDOH Re-engineering Primary Health Care in South Africa 2010 Health Editor, South African National Department of Health: Pretoria

[B24] SANDOH Do. Health National Antenatal Sentinel HIV and Syphilis Prevalence Survey in South Africa, 2009 2010 South African National Department of Health: Pretoria

[B25] PDQ Partnership Defined Quality - A Tool Book for Community and Health Provider Collaboration for Quality Improvement 1999 Save the Children: Washington

[B26] Nkonki L Daviaud E J L A review of costing tools: an exercise to inform a design of a costing tool Inaugural African Health Economics Conference 2009 Accra: Ghana

[B27] WHO Making choices in health: WHO guide to cost-effectiveness analysis 2006 World Health Organization: Geneva

[B28] UNAIDS Costing Guildlines for HIV Prevention Strategies 2000 UNAIDS: Geneva

[B29] Drummond MF eds Methods for the economic evaluation of health care programmes - 3rd edition 2005 Oxford University Press: Oxford

[B30] Robberstad B Economic evaluation of Health interventions in sub-Saharan Africa. Applied economic evaluations and studies on time preferences for health in Tanzania 2005 University of Bergen: Bergen

[B31] Tomlinson M The use of mobile phones as a data collection tool: a report from a household survey in South Africa BMC Med Inform Decis Mak 2009 9 51 10.1186/1472-6947-9-51 20030813 PMC2811102

